# Diagram-based Analysis of Causal Systems (DACS): elucidating inter-relationships between determinants of acute lower respiratory infections among children in sub-Saharan Africa

**DOI:** 10.1186/1742-7622-10-13

**Published:** 2013-12-06

**Authors:** Eva A Rehfuess, Nicky Best, David J Briggs, Mike Joffe

**Affiliations:** 1 MRC-HPA Centre for Environment and Health, Department of Epidemiology and Biostatistics, School of Public Health, Imperial College London, London, UK; 2 Institute for Medical Informatics, Biometry and Epidemiology, University of Munich, Marchioninistrasse 15, 81377 Munich, Germany

**Keywords:** Africa, Children, Acute lower respiratory infections, Pneumonia, Health determinants, Causal diagrams, Multi-factorial causality, Systems epidemiology, Social epidemiology, Environmental epidemiology

## Abstract

**Background:**

Effective interventions require evidence on how individual causal pathways jointly determine disease. Based on the concept of systems epidemiology, this paper develops Diagram-based Analysis of Causal Systems (DACS) as an approach to analyze complex systems, and applies it by examining the contributions of proximal and distal determinants of childhood acute lower respiratory infections (ALRI) in sub-Saharan Africa.

**Results:**

Diagram-based Analysis of Causal Systems combines the use of causal diagrams with multiple routinely available data sources, using a variety of statistical techniques. In a step-by-step process, the causal diagram evolves from *conceptual* based on *a priori* knowledge and assumptions, through *operational* informed by data availability which then undergoes empirical testing, to *integrated* which synthesizes information from multiple datasets. In our application, we apply different regression techniques to Demographic and Health Survey (DHS) datasets for Benin, Ethiopia, Kenya and Namibia and a pooled World Health Survey (WHS) dataset for sixteen African countries. Explicit strategies are employed to make decisions transparent about the inclusion/omission of arrows, the sign and strength of the relationships and homogeneity/heterogeneity across settings.

Findings about the current state of evidence on the complex web of socio-economic, environmental, behavioral and healthcare factors influencing childhood ALRI, based on DHS and WHS data, are summarized in an integrated causal diagram. Notably, solid fuel use is structured by socio-economic factors and increases the risk of childhood ALRI mortality.

**Conclusions:**

Diagram-based Analysis of Causal Systems is a means of organizing the current state of knowledge about a specific area of research, and a framework for integrating statistical analyses across a whole system. This partly *a priori* approach is explicit about causal assumptions guiding the analysis and about researcher judgment, and wrong assumptions can be reversed following empirical testing. This approach is well-suited to dealing with complex systems, in particular where data are scarce.

## 

*“Epidemiology will only progress if it combines a detailed understanding of the ways through which the historical, economic and political constitution of how the world is influences the health of populations—and thus, the individuals within these populations—with the appropriate development of methodology and concepts to deal with this complexity” – George Davey Smith*[1].

*“… a shift in our methodological approach may allow us to better grapple with the complexity of causation within a multilevel understanding of disease etiology.” – Sandro Galea*[2]*.*

## Background

### Childhood acute lower respiratory infections in sub-Saharan Africa

Acute lower respiratory infections (ALRI) represent a permanent global emergency [3]. They were responsible for 6.8% of 2.8 million neonatal deaths, 20.1% of 2.0 million infant deaths and 12.4% of 2.0 million deaths among children aged 1 to 4 years respectively in 2010 [4], with sub-Saharan Africa being the most affected world region [5]. Childhood ALRI is one of several enormous public health problems in developing countries that fail to attract the necessary research to elucidate more of their epidemiology, and how this relates to the environmental and social context.

One of the reasons for this lack of attention is the difficulty of obtaining high-quality health information. In most African countries, vital registration systems are either non-existent or do not reach the whole population, and health surveillance is limited [6,7]. Instead, national and international decision-making heavily relies on nationally representative household surveys that generate cross-sectional datasets that are largely comparable across countries.

Childhood ALRI is the outcome of a web of interacting socio-economic, environmental, behavioral and healthcare factors. Interventions can thus be directed at curbing mortality (e.g. access to healthcare, timely treatment with antibiotics) [8,9], at reducing the risk of infection (e.g. improved nutrition, promotion of breastfeeding, reduced exposure to indoor air pollution) [10-13], or at instigating longer-term socio-economic changes to create healthier societies [14].

Context is crucial. Fewer than 20% of children with ALRI receive appropriate treatment [15]. Even with successful cure, children return to a home with high risk of re-infection. Interestingly, childhood ALRI deaths in the United States fell by two thirds during the first three decades of the 20th century, prior to the introduction of antibiotics or vaccines [3]. This implies that interventions to improve living conditions play a critical role in reducing ALRI morbidity and mortality [16].

### Systems epidemiology

Developing effective interventions requires evidence not only on how individual causal mechanisms influence ALRI risk but also on how these jointly determine disease. This concept could be called systems epidemiology [17]: analysis of the whole system of humans in their total environment within which disease occurs. While systems thinking is increasingly being discussed as important [18,19], a whole-system approach to analysis is still rare in epidemiology.

Causal diagrams provide an important basis for such an approach. Traditional epidemiology is often confined to the analysis of single links between a proximal determinant and disease, and causal diagrams were introduced in this context, mainly to aid causal inference [20,21]. In addition, systems approaches using causal diagrams have been introduced in specific contexts, notably infectious disease modeling using flow diagrams and differential equations [22,23]; causal mediation analysis [24]; and the emerging field of dynamic systems modeling [25,26].

In principle, a systems perspective can also be applied to large and complex causal networks involving environmental or social factors [27] and/or bio-molecular or genetic pathways [17,28,29]. A causal diagram then constitutes a means of organizing the current state of knowledge about that specific area of research, and a framework for integrating statistical analyses [17,28,29]. It consists of all the constituent hypotheses within the system, each represented by a link, plus the additional pathways that are necessary for causal inference (e.g. the potential confounders).

In systems epidemiology, diagram construction involves a partly *a priori* approach: the causal diagram does not emerge from the data but is developed by combining prior knowledge with empirical testing. The former is obtained from pre-existing evidence of various types (e.g. mechanistic or statistical). Thus, constructing such a diagram requires that its structure and content be specified upfront from existing knowledge about the system, supplemented by plausible assumptions where evidence is lacking. It is vital that this includes a complete or near-complete set of potential confounding and selection effects for all the hypotheses embodied in the diagram. Several studies have combined *a priori* conceptualization of health determinants with modeling, using, for example, quantile regression [30], graphical chain models [31-33] or hierarchical models [34]. Other possible statistical tools for estimating causal diagrams include structural equation modeling [24,35,36], propensity score matching [37,38] and instrumental variables [28,39,40].

To our knowledge, all of the studies published to date were based on a single dataset. In systems epidemiology, the causal diagram represents the overall state of knowledge about the system. This means that empirical testing can, and often has to, involve multiple datasets. One advantage is repeated examination, where the same link is explored using more than one dataset, allowing the robustness of the evidence to be assessed. A second is that, for complex systems which cannot be covered by an individual dataset, different parts of the system can be examined using different datasets. This may entail decomposing the diagram into corresponding sub-diagrams. Ideally, this decomposition can be done using conditional independence, so that each relationship or group of relationships becomes testable as an individual hypothesis, without introducing further confounding or other distortions [41], but this is not always possible. Where appropriate, selection effects (colliders) can also readily be introduced into a causal diagram [42].

### Objective

In this paper, we seek to contribute to the concept of systems epidemiology [17] by demonstrating how Diagram-based Analysis of Causal Systems (DACS) can be used to investigate complex questions of disease causation, including both proximal health risks and “causes of causes” [43]. This approach to analysis combines the use of causal diagrams with multiple routinely available data sources, employing a variety of statistical techniques. In the following, we demonstrate the step-by-step progression from a merely plausible *a priori* causal diagram towards one supported by empirical analysis, aimed at deciphering the contributions of proximal and distal risk factors to childhood ALRI.

## Methods

This section provides an overview of the steps of causal diagram development (Table 1 shows the specific terminology to describe this process), gives a detailed account of the data used and statistical approach employed, and describes transparent criteria for testing causal diagrams.

**Table 1:** Terminology for key steps in Diagram-based Analysis of Causal Systems

**Type of diagram**	**Definition**	**Use**
***Conceptual causal diagram***	A causal diagram developed using *a priori* knowledge, and supplemented by assumptions where necessary. This describes the whole system, and includes all potentially important proximal and distal determinants of a health outcome, potential pathways connecting them, and potential confounders and/or selection effects. All variables are in their conceptual or ideal form.	A conceptual causal diagram provides the underlying conceptual framework for statistical analysis, independent of the specific statistical approach or approaches chosen.
***Operational causal diagram***	A causal diagram derived from the conceptual diagram, and informed by data availability as determined by one data source. All variables are in their actually measured form, which may include proxies depending on the data source. Pathways connecting them may be testable (where all relevant variables are measured) or conceptual (where some or all relevant variables are unmeasured). Depending on the available data and approach to analysis, distinct versions of the operational causal diagrams may be developed for different data sources or datasets* (e.g. for different countries, settings or years).	An operational causal diagram provides the basis for statistical analysis, shedding light on specific statistical approaches that may be applied to a given data source.
***Integrated causal diagram***	A causal diagram derived from the conceptual diagram, as informed by empirical testing across more than one data source. As for operational causal diagrams, variables and the pathways connecting them may be in their actual or conceptual form; actual variables and the pathways connecting them derive from more than one data source.	An integrated causal diagram provides a current summary of knowledge about the whole system, illustrating causal pathways that are well-supported *versus* causal pathways where evidence is lacking.

* We use “data source” to refer to different types of data (e.g. DHS vs. WHS data), and “dataset” to refer to the same type of data being available for different settings (e.g. DHS data for Benin vs. DHS data for Ethiopia).

### Steps of causal diagram development

**Figure 1:**
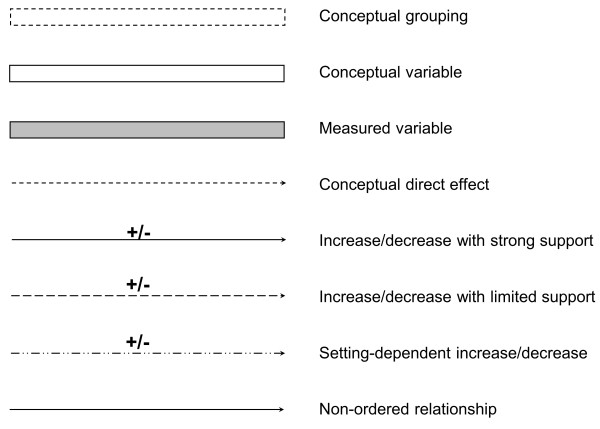
Notational conventions.

The approach follows the notational conventions of Earp and Ennett [44], Robins [21] and Best and Green [45] (see Figure 1). It relies on the initial assumptions that causal relationships apply independently of the data source used to assess them; and that they are stable between populations and over time. These assumptions can be tested empirically and rejected if appropriate. The timeframe must be clearly specified, as must the unit of analysis (i.e. individual vs. aggregate) and level of aggregation or spatial scale, as they influence the identification and definition of variables included. We focus on relatively short-term processes affecting health outcomes among children under five years of age.

Step 1: a *conceptual causal diagram* was constructed *a priori* based on state-of-the-art knowledge, independent of data availability (Table 1). This was organized into hierarchical layers, which represent the causal and temporal ordering of the different groups of variables (we prefer the term “layer” to “level”, as the latter could be confused with its quite different usage in the context of multilevel modeling). A four-layer diagram resulted, with layers representing context, household socio-economic status, proximal health risks and health outcomes; as a convention, health outcomes are depicted as being harmful [27]. Specificity was introduced by attempting to identify *all* distinct variables in each layer and *all* potential causal pathways that connect variables within and across layers, including potential confounders.

This diagram resembles a graphical chain model [46,47] in the sense that its variables are partitioned into hierarchically arranged subsets (i.e. conceptual groupings), with arrows representing causal associations linking variables in adjacent subsets. Within these conceptual groupings associations between variables are assumed to be non-causal. We represent this assumption by the absence of arrows (e.g. no arrow connecting the different variables within material situation), whereas in graphical chain models, non-causal associations (correlations) between variables within a subset are empirically tested and represented by undirected links.

The omission of an arrow is a stronger decision than its inclusion, because specifying a link that does not exist in the real world will be discovered by finding a parameter value of zero, whereas once omitted the link cannot be tested. In other words, *a priori* inclusion is more conservative than omission – i.e. it errs on the side of caution – as any false assumptions can still be rectified after empirical testing.

Step 2A: data availability informed which parts of this diagram could be examined using a specific dataset. Conceptual variables were replaced with measured variables, resulting in an *operational single-dataset causal diagram* (see Table 1). As omission of relevant variables and relationships, where these cannot be measured, is likely to result in residual confounding, conceptual variables and the paths connecting them were retained in the diagram. This serves to ensure that all relevant common causes and colliders that could affect a particular path between exposure and outcome are included. Where this is the case, each arrow in the operational single-dataset causal diagram represents a separate hypothesis. These were tested – in our case using a Demographic and Health Survey (DHS) dataset for Benin – to assess overall support for the hypothesis (see Data and statistical analysis and Criteria for testing causal diagrams).

Step 2B: testing across multiple similar datasets generated an *operational multiple-dataset causal diagram* (see Table 1) in our case using DHS datasets for Kenya, Ethiopia and Namibia. This step has a confirmatory function, the aim being to assess the degree of consistency/robustness of the step 2A diagram across populations. In making these comparisons, attention must be paid to selection factors or colliders which may differ between populations; these could be examined explicitly [42] and, where appropriate, included in country-specific operational causal diagrams. Hypothesis testing was repeated and conclusions were drawn about the sign of the relationships in each hypothesis (i.e. a decreasing or increasing function), and about its strength (i.e. strong or limited). Where a given path between measured variables did not show any effect across settings, the path was removed from the diagram (see Data and statistical analysis and Criteria for testing causal diagrams).

Step 3: as a single type of dataset rarely includes all the information required for testing a whole conceptual diagram, other datasets with a complementary function can be brought in. We used a pooled World Health Survey (WHS) dataset to examine the pathway linking socio-economic status, solid fuel use and ALRI mortality. The results obtained from WHS testing and DHS testing were combined in an *integrated causal diagram* (see Table 1). This represents our current best understanding of the relationships specified in the original conceptual diagram, but it is not in any sense a final version; such diagrams continue to evolve as more evidence becomes available to confirm, extend and refine the operational version. This involves repeating steps 2 and/or 3, using the most appropriate statistical approaches in view of data availability and the strength and consistency of evidence already represented in the diagram. Suitable methods include regression techniques, structural equation modeling and instrumental variable approaches, depending on the nature of the relationships to be examined and the data available; Bayesian approaches and/or various types of meta-analysis [48] can also be used to formally combine new evidence with the existing evidence represented in the diagram. Also, periodic reassessment of the adequacy of the original conceptual diagram may be necessary (step 1).

### Data and statistical analysis

We used DHS [49] and WHS data [50], nationally representative household surveys with response rates well above 90%, that provide high-quality and comparable information on health and risk factors for a large number of developing countries. DHS datasets for Benin, Kenya, Ethiopia and Namibia were selected because they included information on the main cooking fuel used; they were analyzed separately to allow for a distinction between those causal links that are likely to be consistent between populations and those that may operate in a population-specific fashion (Table 2). WHS data for sixteen countries of sub-Saharan Africa were pooled (Table 3; [51]).

**Table 2:** DHS sample sizes by country

**Country**	**# households (response rate)**	**# women of reproductive age (response rate)**	**# children <5 years of age**
**Benin**	5,769 (97.0)	6,219 (96.4)	4,597
**Ethiopia**	14,072 (99.3)	15,367 (97.8)	9,255
**Kenya**	6,333 (96.3)	8,195 (94.0)	5,288
**Namibia**	4,653 (96.9)	6,755 (92.4)	3,616

**Table 3:** WHS sample sizes by country

**Country**	**# households (response rate)**	**# women of reproductive age (response rate)**	**# women of reproductive age with children born during last ten years**	**# children born during last ten years**
**Burkina Faso**	5,046 (98)	2,122 (99)	1,629	3,602
**Chad**	5,075 (95)	1,909 (97)	1,054	2,346
**Comoros**	1,860 (98)	636 (97)	313	698
**Congo**	3,158 (64)	1,193 (98)	553	971
**Cote d’Ivoire**	3,298 (81)	1,154 (99)	586	1,052
**Ethiopia**	5,131 (96)	2,053 (99)	1,355	3,260
**Ghana**	5,662 (73)	1,529 (97)	991	2,047
**Kenya**	5,365 (81)	1,985 (96)	1,447	3,197
**Malawi**	5,727 (93(	2,396 (96)	1,785	3,856
**Mali**	5,445 (94)	1,270 (85)	351	827
**Mauritania**	3,929 (95)	1,754 (99)	767	1,656
**Namibia**	4,656 (93)	2,025 (99)	1,175	2,034
**Senegal**	3,649 (69)	1,205 (90)	402	809
**Swaziland**	3,122 (54)	1,323 (98)	480	956
**Zambia**	4,350 (83)	1,794 (94)	1,268	2,898
**Zimbabwe**	4,343 (89)	2,096 (99)	1,359	2,411
**Pooled dataset**	69,816	26,444	15,515	32,620

While the DHS provides detailed information on the recent occurrence of childhood illnesses as well as child mortality rates, the WHS assesses child mortality and symptoms prior to death. Variables representing ALRI determinants also vary between the data sources (Table 4).

**Table 4:** Variables related to ALRI and its determinants in DHS and WHS

	**Socio-economic status**	**Risk factor exposure**	**Health outcome**
Household	Paternal education	**Cooking fuel**	
	Paternal occupation	*Cooking stove*	
	**Urban/rural location**	*Cooking location*	
	Household assets*	Overcrowding	
Woman	**Maternal education**	**Maternal smoking**	
	**Maternal occupation**		
Child		Birth weight	ALRI morbidity (cough and rapid breathing during last two weeks)
		Breastfeeding	
		Malnutrition	*ALRI mortality (cough and rapid breathing or chest indrawing prior to death)*
		Micronutrient intake	
		Vaccination status	

Bold: DHS and WHS.

Regular: DHS only.

Italics: WHS only.

* Electric goods, shelter and mobility indices in DHS and WHS; wealth quintiles in WHS only.

Given the characteristics of the hypotheses, we used logistic regression, ordered logistic regression and survival analysis to test individual hypotheses of different outcomes [52,53]; survival analysis was also used to examine the impact of cooking-related parameters on ALRI mortality [51]. In this way, empirical evidence (or lack thereof) was assessed for every arrow connecting two measured variables in the operational causal diagrams. In view of non-independence due to children born to the same mother, all analyses were adjusted for clustering to produce robust standard errors, resulting in wider confidence intervals. We did not adjust for stratification and cluster samples and omitted sample weights, a strategy considered conservative for multivariable analysis of household survey data [54]. Models were run in Stata Special Edition 9 for the whole dataset, and separately for urban and rural settings; exploratory analyses, confirmed by cluster analysis, had revealed striking urban–rural differences in all countries [55].

For each hypothesis, the full model including all measured explanatory variables and all possible nested models were run. Initially, provided the univariate model explained the data significantly better than the null model with p < 0.05, all variables were retained. Subsequently, nested models were compared against the full model using the Akaike Information Criterion (AIC) to explore whether different explanatory variables contributed independently to the outcome of interest. Only differences of 3 or more points are considered meaningful in terms of indicating better model fit [56]; for differences of less than 3 points, the more complete model was reported in line with a conservative approach. Where the diagram showed unmeasured explanatory variables, their likely influence in the form of residual confounding was carefully considered in the interpretation of findings.

### Criteria for testing causal diagrams

As we relied on different data sources to inform different parts of the diagram, we decomposed it into a series of separate hypothesis tests and, similar to Weng and colleagues [57], employed conventional covariate selection techniques for testing. The criteria described here provide a basis for replicating the analysis, conducting sensitivity analyses and incorporating new datasets and data sources as they become available. They were developed in an attempt to minimize the role of researcher judgment, and where this is unavoidable make its use explicit and transparent; they represent but one way of doing so.

As inclusion is more conservative than omission, it is important to ensure that no hypothesis is rejected prematurely because of a country-specific situation or data quality issues. Therefore, in step 2A, if any of the univariate models explained the data significantly better than the null model in at least one of the three settings, this was considered to provide evidence towards the hypothesis. This initial testing stage only assessed overall support for a given hypothesis involving several variables, not the role of individual variables.

**Figure 2:**
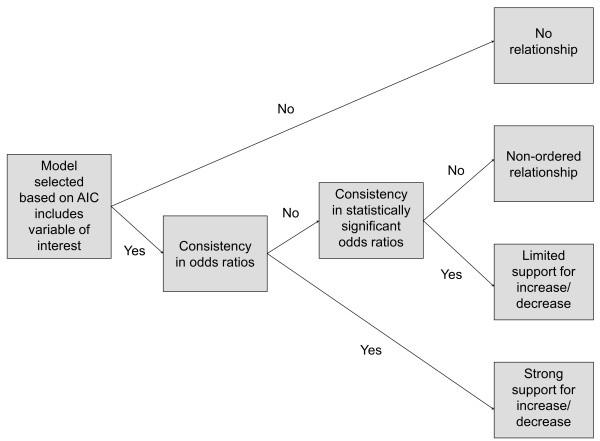
**Assessment of sign of relationship in individual settings.** Consistency in (statistically significant) odds ratios is determined by assessing whether the central estimates of (statistically significant) odds ratios across different levels of a given variable (e.g. low, intermediate, high electric goods index) are always above or always below 1.

Figure 2 depicts the decision-making process to assess the sign of a relationship in individual settings. For any hypothesis tested in step 2B, each explanatory variable was considered in turn and separately for urban and rural settings; only variables included in the model selected based on the AIC were judged to have a direct and at least partly independent effect on the outcome variable. Absolute sample sizes, relative sample sizes (i.e. small sub-groups with respect to variables of interest) and measurement error can all affect whether a true relationship is measured as statistically significant or not. We therefore contend that consistency (i.e. central estimates of odds ratios are always above or always below 1) across different levels of a given variable (e.g. low, intermediate, high electric goods index) is potentially a more reliable guide for assessing the sign of a relationship than statistical significance. As detailed in Figure 2, this consistency criterion was applied to distinguish between strong *versus* limited support for an increasing or decreasing effect on the outcome variable, or to conclude that the relationship is non-ordered.

Once the assessment on a country-by-country and setting-by-setting basis was completed, a judgment needed to be made about homogeneity/heterogeneity. Three situations put consistency at stake:

➢ A relationship is classified as increasing across all levels of a variable in some settings but absent in the remaining settings. Such findings contradict consistency but may be explained by small differences in causal mechanisms or by random error.

➢ A relationship is classified as increasing across all levels of a variable in some settings but as decreasing in the remaining settings. Such findings strongly contradict consistency as they point towards distinct setting-specific causal mechanisms unlikely to be explained by chance.

➢ A relationship is classified as non-ordered (i.e. showing an effect, with the sign of the relationships varying at different levels of a variable) in some but as increasing in other settings. Consistency can easily accommodate findings that go further in a sub-set of settings relative to the rest by “downgrading” the classification of the relationship to non-ordered.

**Figure 3:**
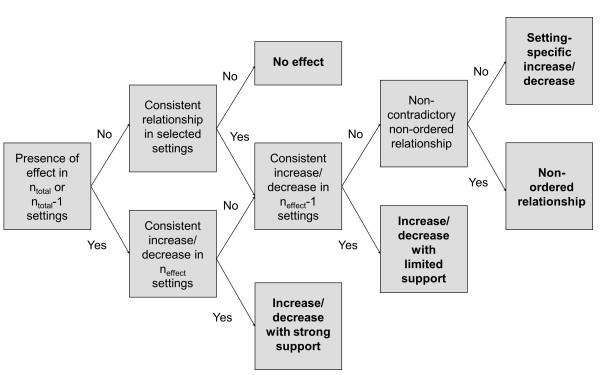
**Assessment of homogeneity/heterogeneity across settings.** n_total_ refers to all settings where the hypothesis can be tested. n_effect_ refers to all settings where an effect can be detected.

In deriving an operational multiple-dataset causal diagram in step 2B, we distinguish between an increasing/decreasing function with strong or limited support, a non-ordered relationship, a setting-specific increasing or decreasing function and no effect, taking into account the possibility of “false negatives” due to measurement error, other data quality issues or random error. As illustrated in Figure 3, we made the somewhat arbitrary decision to allow for one exception regarding the presence or absence of an effect (n_total_-1) and the sign of a relationship (n_effect_-1). We only considered there to be sufficient evidence of a null effect, when an effect was tested across multiple settings and no relationship was observed in any of these; only under these circumstances was an arrow removed from the diagram.

## Results

Rather than giving detailed results for each hypothesis, this section summarizes the overall findings; Table 5 provides a detailed description of the step-by-step process of causal diagram development and testing for one hypothesis; a similar account for all other hypotheses can be found in Rehfuess [55].

**Table 5:** A step-by-step example: impact of wealth and parental education on solid fuel use

***Step 1: An a priori conceptual causal diagram based on state-of-the-art knowledge***	Few published studies have quantitatively assessed the determinants of cooking practices [58,59]; two systematic reviews of peer-reviewed quantitative studies [60] and peer-reviewed qualitative studies as well as grey literature [61] regarding factors that influence household adoption of cleaner fuels and improved cookstoves are currently underway. Based on this limited evidence base, we postulate that cooking fuel use is influenced by: • Wealth, through financial access to cleaner fuels or more efficient, cleaner-burning stoves; • Maternal and paternal education, through knowledge about the health risks associated with indoor air pollution and prioritization of resources towards solving this problem. • This *a priori* statement is graphically depicted in Figure 4 and summarized as evidence-based hypothesis 9 (Table 6).
***Step 2A: An operational single-dataset causal diagram using a DHS dataset for Benin***	As the rural Beninese population almost exclusively relies on solid fuels, hypothesis 9 could only be tested in urban Benin. Univariate and multivariable logistic regression analyses show consistent trends in odds ratios for wealth, maternal education and paternal education (Table 7). These findings provide support for the stated hypothesis, leading to retention of all three arrows in the operational single-dataset causal diagram (Figure 5).
***Step 2B: An operational multiple-dataset causal diagram using DHS datasets for Kenya, Ethiopia and Namibia***	Equally, the analysis in Ethiopia, Kenya and Namibia concludes that all three socio-economic factors play a role (Table 7). Applying the testing criteria in Figure 2, greater maternal education consistently shows odds ratios below 1 in all six settings, where the hypothesis could be tested, implying strong support in individual settings. Applying the testing criteria in Figure 3, it can be concluded that maternal education decreases the chances of solid fuel use with strong support, which graphically translates as a solid decreasing arrow in the operational multiple-dataset causal diagram (Figure 6). Paternal education, on the other hand, was not part of the model selected by the AIC in rural Kenya and urban Namibia; testing in the other four settings concluded with limited (urban Ethiopia, urban Kenya) or strong (urban Benin, rural Namibia) support for a decreasing relationship. Paternal education may therefore not exert an independent influence everywhere, leading to the inclusion of a setting-specific decreasing arrow. Wealth emerges as important across all six settings; in Namibia, however, better mobility – as one dimension of wealth – increases the chances of solid fuel use, a finding that turns out to be robust in several sensitivity analyses. Based on the testing criteria in Figure 3, a setting-specific decreasing arrow best captures the relationship between wealth and solid fuel use (Figure 6).
***Step 3: An integrated causal diagram using a pooled WHS dataset for sixteen African countries***	With the exception of paternal education, all variables relevant to hypothesis 9 are available in DHS and WHS and assessed in a comparable way; their population distribution in Ethiopia, Kenya and Namibia is similar. Hypothesis testing confirms the robustness of the links between wealth, maternal education and solid fuel use in the individual WHS datasets, as well as in the pooled WHS dataset (Table 8). Consequently, the relationships derived for Figure 6 are incorporated in the integrated causal diagram (Figure 7).

**Figure 4:**
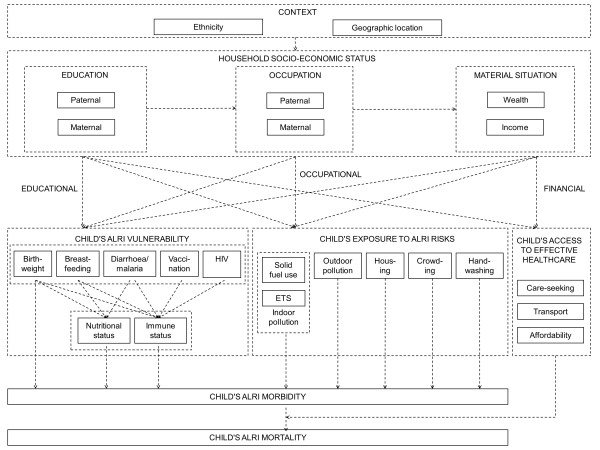
A conceptual causal diagram.

The conceptual causal diagram in Figure 4 (step 1) summarizes how distinct socio-economic determinants (embedded in a given national or local context) and proximal health determinants (grouped as vulnerability, exposure and access to effective healthcare) jointly determine a child’s risk of ALRI morbidity and ALRI mortality. Based on *a priori* knowledge derived from a review of the literature and assumptions, it is a graphical representation of our conceptualization of reality which, given an ideal dataset, could be examined in its entirety.

Using the Beninese dataset, “conceptual variables” in Figure 4 were replaced with actual variables. The DHS does not provide cause-of-death information, making an assessment of ALRI mortality impossible. A significant challenge in examining ALRI morbidity among children is the distinction between frequent but harmless infections of the upper respiratory tract and rare but potentially life-threatening infections of the lower respiratory tract. Preliminary analysis of DHS records of cough and fast breathing during the two weeks prior to the survey indicated that they could not serve as a useful proxy for ALRI. Consequently, impacts on the health layer (i.e. child’s ALRI morbidity and mortality) could not be investigated in the operational single-dataset causal diagram (step 2A). As child’s access to effective healthcare operates by influencing progression from morbidity to mortality, the effect of healthcare-related variables could also not be tested.

**Figure 5:**
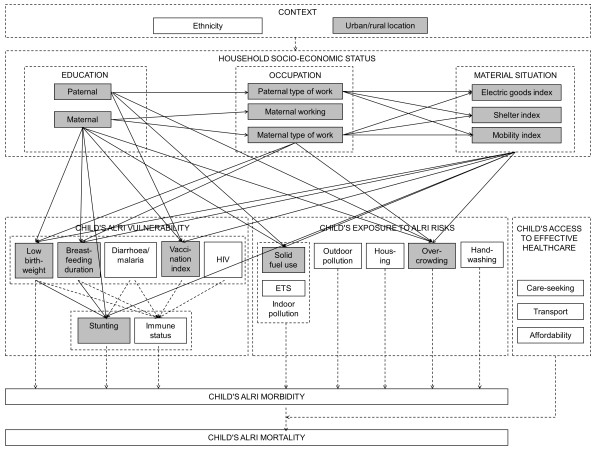
An operational single-dataset causal diagram.

The DHS assesses a large number of socio-economic and proximal determinants of child ALRI (Table 4), allowing us to populate many of the originally specified conceptual variables (Figure 5). For some, however, no suitable proxy was available. For example, information on maternal smoking was available for all children in the DHS datasets and selected children in the WHS datasets. In the sub-Saharan countries examined here, however, maternal smoking rates are very low and information was not available about the smoking habits of other household members. Maternal smoking was therefore considered an inadequate measure of environmental tobacco smoke and was not examined further. Independent hypotheses based on measured variables were formulated (Table 6). Causal diagram testing produced evidence in support of all hypotheses in Benin (Figure 5; [55]).

**Table 6:** Empirically testable hypotheses

**Relations between different socio-economic factors (DHS, WHS)**	
Hypothesis 1	Paternal education impacts on paternal occupation.
Hypothesis 2	Maternal education impacts on maternal occupation.
Hypothesis 2a	Maternal education impacts on maternal occupation (working/not working).
Hypothesis 2b	Maternal education impacts on maternal occupation (type of work).
Hypothesis 3	Paternal and maternal occupations impact on household wealth.
Hypothesis 3a	Paternal and maternal occupations impact on the electric goods index.
Hypothesis 3b	Paternal and maternal occupations impact on the shelter index.
Hypothesis 3c	Paternal and maternal occupations impact on the mobility index.
**Relations between different proximal health risks (DHS)**	
Hypothesis 4	Low birthweight and breastfeeding duration impact on stunting [62-65].
**Socio-economic factors as determinants of proximal health risks (DHS, WHS)**	
Hypothesis 5	Wealth, maternal education and maternal occupation impact on low birthweight [66].
Hypothesis 6	Wealth, maternal education and paternal education impact on stunting [67-71].
Hypothesis 7	Wealth, maternal education and maternal occupation impact on breastfeeding duration [72].
Hypothesis 8	Wealth, maternal education and paternal education impact on vaccination index [73,74].
Hypothesis 9	Wealth, maternal education and paternal education impact on solid fuel use [58,59].
Hypothesis 10	Wealth, maternal education, maternal occupation and paternal education impact on overcrowding.
**Socio-economic and proximal health risks as determinants of ALRI mortality (WHS)**	
Hypothesis 11	Maternal education, wealth and solid fuel use impact on child’s ALRI mortality.

**Figure 6:**
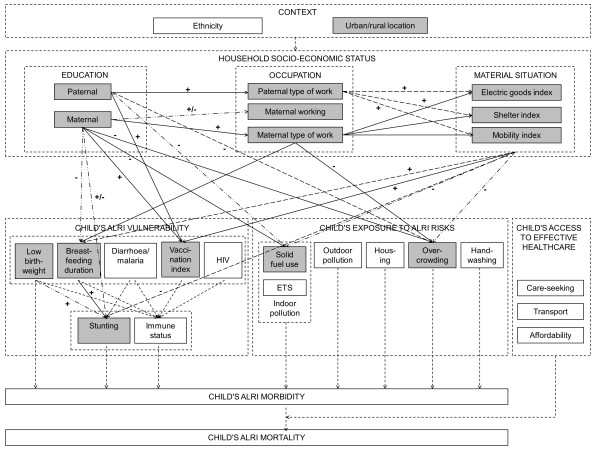
An operational multiple-dataset causal diagram.

Figure 6 summarizes results of step 2B. We found that education, occupation and wealth exert their influence on proximal health risks through at least partly independent pathways, and that some dimensions of socio-economic status, in particular material circumstances and related purchasing power, play a greater role in determining risk factor profiles than others. Stunting, solid fuel use and vaccination emerge as particularly strongly structured by socio-economic variables.

The relationships among socio-economic factors (hypotheses 1–3) and among proximal health determinants (hypothesis 4) show a high degree of consistency and appear to apply independent of country or geographical setting. More heterogeneity is observed for the impact of socio-economic factors on proximal health determinants (hypotheses 5–10). This is due in part to variation in the relative importance of a given measure of socio-economic status, and in part to different mechanisms operating in different settings. The latter highlights the importance of contextual factors, such as ethnicity and urban–rural location. In the present analysis, we used contextual factors (i.e. urban–rural location) as stratification variables. As all variables identified for household socio-economic status identified in the conceptual diagram were populated, unmeasured confounding is negligible, provided the diagram was correctly specified.

The WHS assesses several socio-economic determinants, solid fuel use and, importantly, cause-specific child mortality (Table 4), and can therefore be used in step 3. Here we examined how maternal education, wealth and solid fuel use jointly influence ALRI mortality (hypothesis 11); the explanatory variable paternal education was not available in the WHS but, given its high correlation with maternal education, residual confounding is likely to be minor. We used an *a priori* ALRI definition of cough accompanied by rapid breathing or chest indrawing that closely resembles the ALRI algorithm in standard verbal autopsy tools [75]. First, robustness of the links between wealth, maternal education and solid fuel use was assessed in individual countries and in the pooled African dataset (Tables 5, 7 and 8), indicating a high degree of compatibility between DHS and WHS. Subsequently, determinants of ALRI mortality were examined in the pooled African dataset.

**Table 7:** Results for hypothesis 9 using DHS data: Odds ratios for logistic regression of solid fuel use on wealth, maternal education and paternal education**

	**Benin**		**Ethiopia**		**Kenya**		**Namibia**		**Conclusion**
	**Urban**	**Rural**	**Urban**	**Rural**	**Urban**	**Rural**	**Urban**	**Rural**	
**Electric goods**									
**low**	6.30*	N/A	1.00	N/A	1.00	1.00	1.00	27.43*	
**intermediate**	1.00	N/A	0.21*	N/A	0.47*	0.25*	0.31*	1.00	
**high**	0.53*	N/A	0.09*	N/A	0.20*	0.02*	0.04*	0.07*	
**Shelter**									
**low**	1.97	N/A	23.77*	N/A	1.00	3.58	N/A	N/A	
**intermediate**	1.00	N/A	1.00	N/A	0.63	1.00	N/A	N/A	
**high**	-	N/A	0.30*	N/A	0.52*	0.36*	N/A	N/A	
**Mobility**									
**low**	1.00	N/A	1.00	N/A	1.00	1.00	1.00	1.00	
**intermediate**	1.50	N/A	0.47*	N/A	0.69	0.78	1.69*	2.28*	
**high**	1.14	N/A	-	N/A	-	-	-	-	
	Decrease	N/A	**Decrease**	N/A	**Decrease**	**Decrease**	Non-ordered	Non-ordered	Setting-specific decrease
**Maternal education**									
**none**	1.38	N/A	1.00	N/A	1.00	1.00	1.00	1.00	
**primary**	1.00	N/A	0.72*	N/A	0.41*	1.00	0.48*	0.79	
**secondary**	0.56*	N/A	0.68*	N/A	0.39*	0.86	0.20*	0.32*	
**higher**	-	N/A	-	N/A	0.44*	0.49	0.10*	0.52	
	**Decrease**	N/A	**Decrease**	N/A	**Decrease**	**Decrease**	**Decrease**	**Decrease**	Decrease with strong support
**Paternal education**									
**none**	1.00	N/A	1.00	N/A	8.22*	-	-	7.96*	
**primary**	0.68*	N/A	0.93	N/A	1.00	-	-	1.00	
**secondary**	0.55*	N/A	0.84	N/A	0.89	-	-	0.74	
**Higher**	-	N/A	-	N/A	1.27	-	-	0.47	
	**Decrease**	N/A	Decrease	N/A	Decrease	No effect	No effect	**Decrease**	Setting-specific decrease

* Statistically significant with p < 0.05.

** Bold font indicates strong support, normal font indicates limited support for the direction of the relationship.

**Table 8:** Results for hypothesis 9 using WHS data: Odds ratios for logistic regression of solid fuel use on wealth and maternal education**

	**Ethiopia**		**Kenya**		**Namibia**		**16 countries**	
	**Urban**	**Rural**	**Urban**	**Rural**	**Urban**	**Rural**	**Urban**	**Rural**
**Electric goods**								
**low**	1.00	N/A	1.00	1.00	1.00	10.00*	1.00	1.00
**intermediate**	0.15*	N/A	0.41*	0.12*	0.44*	1.00	0.30*	0.18*
**high**	-	N/A	0.52	-	0.07*	0.14*	0.12*	0.04*
**Shelter**								
**low**	0.20	N/A	1.00	3.06	1.00	4.47*	1.00	1.00
**intermediate**	1.00	N/A	0.41	1.00	0.33*	1.00	0.52*	0.25*
**high**	0.51	N/A	0.52	0.73	0.17*	0.54	0.29*	0.12*
**Mobility**								
**low**	N/A	N/A	1.00	1.00	1.00	1.00	1.00	1.00
**intermediate**	N/A	N/A	0.63	1.64	0.64	1.89	0.52*	0.52*
	Decrease	N/A	Decrease	Decrease	**Decrease**	Decrease	**Decrease**	**Decrease**
**Maternal education**								
**none**	1.00	N/A	1.00	1.00	-	1.00	1.00	1.00
**primary**	0.18*	N/A	0.54	0.81	-	0.81	0.74*	1.14
**secondary**	0.11*	N/A	0.29*	0.35	-	0.22*	0.46*	0.64*
**Higher**	-	N/A	-	-	-	-	0.29*	0.56*
	**Decrease**	N/A	**Decrease**	**Decrease**		**Decrease**	**Decrease**	Decrease

* Statistically significant with p < 0.05.

** Bold font indicates strong support, normal font indicates limited support for the direction of the relationship.

**Figure 7:**
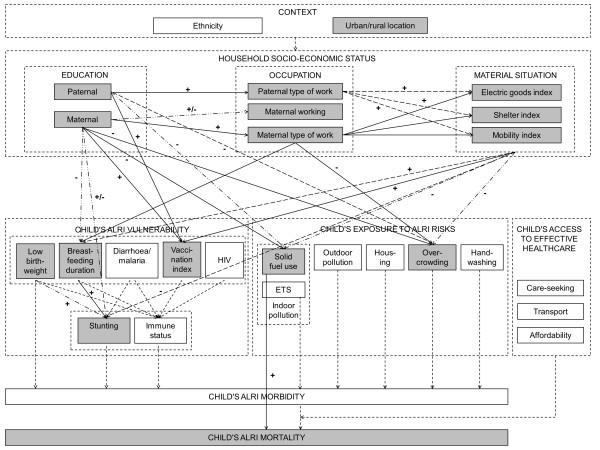
An integrated causal diagram.

In univariate models using the *a priori* ALRI definition, a child’s risk of dying from ALRI prior to its fifth birthday was 2.54 times higher (95% confidence interval: 1.42, 4.55) for children living in households that cook with wood, dung or charcoal relative to children living in households that cook with gas or electricity. High wealth compared to low wealth and secondary or higher education of the mother compared to no education showed ALRI mortality hazard ratios of 0.68 (0.46, 1.00) and 0.65 (0.40, 1.06) respectively. Including wealth and maternal education variables in the solid fuel use model yielded an adjusted hazard ratio of 2.35 (1.22, 4.52). Alternative definitions of ALRI, which were less or more sensitive, resulted in adjusted hazard ratios of 2.07 (1.22, 3.53) and 3.21 (1.18, 8.76) respectively [51]. Based on these findings an arrow representing an increasing relationship with strong support was introduced to link solid fuel use and ALRI mortality. This integrated causal diagram summarizes our current understanding of the web of ALRI determinants (Figure 7).

## Discussion

### Strengths and weaknesses of Diagram-based Analysis of Causal Systems

The basic idea of DACS is the use of causal diagrams as a formal tool for representing state-of-the-art knowledge of a specific research area. This whole-system approach makes the analyst’s view of reality and assumptions explicit; generates testable hypotheses; provides a framework for model building and statistical analysis, potentially using a broad range of statistical methods; accommodates multiple sources of evidence; evolves from conceptual to empirically supported; identifies research gaps; highlights entry-points for public health interventions; and facilitates communication between stakeholders.

Constructing a causal diagram involves a dialog between an *a priori* statement of causal structure and an inductive *a posteriori* method of testing. Each informs the other: the structure facilitates design of studies to test specific hypotheses, and accumulating evidence stimulates revision of the causal structure. Each diagram explicitly represents not only pathways of substantive interest, but also those that could bring about non-random statistical associations in the absence of direct causation: i.e. reverse causation, confounding (common ancestor(s)) or Berksonian bias (common descendant(s)). By representing all relevant potential causal pathways, this resembles established epidemiological methodology of directed acyclic graphs for addressing biases and selection effects relating to a single causal pathway [20,21,44,76,77].

While individual causal pathways in our approach resemble directed acyclic graphs, the overall DACS approach is more closely related to graphical chain models, where complex association structures are explicitly recognized, sets of variables are placed along a dependence chain and then modeled using a series of regression analyses [46,47]. DACS goes beyond conventional graphical chain models, in particular by: (i) highlighting the importance of evidence in developing the initial conceptual diagram, which leads to a more finely-structured diagram comprising conceptual groupings within layers (=blocks) rather than only blocks and which explicitly promotes a hypothesis-driven analysis strategy; (ii) emphasizing model building through a structured step-by-step method, and embracing a forward-looking approach, where the causal diagram is expected to continue to evolve over time; and (iii) allowing for the possibility of drawing on multiple datasets, both for the original analysis and for the subsequent addition of information.

The last point in particular is a key strength of DACS and carries two advantages. First, repeated assessment allows the robustness of a single causal pathway across data sources and settings to be examined. Secondly, following decomposition of the system, relevant sub-structures can be assessed using different data sources, although this does entail specifying potential confounders that may be introduced where conditional independence cannot be used.

The requirement to specify *all* possible causal pathways, including those that cannot be observed, is formidable both with a causal diagram-based and the traditional approach. Researcher judgment plays a role in both, but is more explicit in the combined *a priori*/empirical method because it is exercised prior to the analysis, and diagramming makes it visible and transparent. With a purely inductive approach, findings that are not based on previously specified hypotheses may be easier to ignore or dismiss when inconvenient [77], and such findings are suspect when novel – the “fishing expedition” problem. With a partly *a priori* approach, judgments and assumptions are uninfluenced by the data and have to be made transparent; any changes following the statistical analysis must be explicitly stated and justified. Indeed, incorrect researcher judgment is also potentially reversible: the conceptual diagram is periodically revised as further evidence becomes available. Initial conceptual diagrams are, however, not necessarily unique, and it is good practice for researchers to use their diagrams to explore underlying differences in their assumptions [27]. Importantly, it is impossible to specify a correct analytical strategy without causal knowledge or assumptions [57,76,78].

### Strengths and weaknesses of the application in this study

The application of this method to the problem of ALRI in developing countries here shows several specific weaknesses. In most complex systems there are likely to be some unspecified or unmeasured common causes, and this almost certainly holds true for the present analysis, which was limited by data availability and quality [55]. Neither the DHS nor the WHS include all relevant variables and, as a result, we were unable to control adequately for confounding in several of the observed relationships. Moreover, several variables are subject to significant measurement error (e.g. a household’s main cooking fuel as proxy for a child’s exposure to indoor air pollution in DHS and WHS; a combination of reported symptoms prior to child death as proxy for clinically-confirmed cause of death in WHS).

In addition, the cross-sectional nature of the datasets precluded analysis of the temporal sequence of events. We carefully assessed the likely impact of this limitation, and consider that reverse causation and feedback loops were unlikely to be important given the short timeframe of interest specified.

Another limitation is that our models assume simple (e.g. linear) functional form and an absence of effect modification or statistical interaction. With the future availability of more and richer datasets on ALRI determinants, it will become possible to test, and if necessary modify, this assumption. Finally, the countries investigated here represent a convenience sample – they were not chosen for their representativeness of the African continent. Nevertheless, based on their geography, climate and distinct colonial histories, Benin, Ethiopia, Kenya and Namibia can be considered representative of a variety of living conditions in sub-Saharan Africa, and so can the pooled WHS dataset of sixteen countries. In summary, the results presented here have only interim status, reflecting our understanding of the system based on currently available evidence.

### Key findings and their implications

Our research has only been able to shed light on one of the many pathways that link distal and proximal determinants of childhood ALRI, the inter-linkages between socio-economic status, solid fuel use and ALRI mortality.

The risk of dying from ALRI during childhood varies between population groups but socio-economic differences may be less marked than those commonly observed with respect to all-cause child and infant mortality [79-87]; indeed most of the observed socio-economic gradients in ALRI mortality do not reach significance. This is consistent with previous reports of minor socio-economic gradients in childhood ALRI mortality [88,89] and morbidity [58,90-93].

Wealth, maternal education and, to a lesser extent, paternal education are highly protective against solid fuel use. This suggests that these play at least partly independent roles in structuring cooking fuel use in sub-Saharan Africa. Previous reports [58,59] that cooking with liquefied petroleum gas is more prevalent among sub-populations with higher income and higher educational attainment accord with these findings.

Our research confirms solid fuel use as a major risk factor for ALRI among African children, suggesting that exposure more than doubles the risk of ALRI mortality. These results are comparable to findings in two case–control studies of ALRI mortality conducted in Tanzania [89] and the Gambia [88], and a case–control study of all-cause child mortality in India [94]. Reducing solid fuel use could therefore be an important means of reducing ALRI mortality.

We found that socioeconomic variables affected solid fuel use, and that solid fuel use affected ALRI mortality. For solid fuel use to qualify as a mediator of the impact of socio-economic status on ALRI mortality, the relationship between maternal education/wealth and ALRI mortality should be attenuated once solid fuel use is included in the model [95]. Surprisingly, this was not the case. Common concerns in mediation analysis are unmeasured confounding and measurement error, but in this instance a third issue proved to be the explanation: heterogeneity in the population. Detailed investigations revealed the presence of a threshold effect in the wealth-fuel use relationship: i.e. differences in fuel use are limited among the lower wealth quintiles but stark between the bottom four quintiles and the richest quintile. Within the richest quintile solid fuel use acts as a mediator of the wealth-ALRI mortality relationship: when the variable is included in the model the hazard ratio is attenuated from 0.66 (0.41; 1.07) to 0.87 (0.52; 1.44). Causal diagrams therefore play an important role in making complex issues of mediation and non-transmission apparent.

The integrated causal diagram in Figure 7 illustrates two potential entry-points for interventions. Increasing household wealth and, to a lesser extent, improving education and knowledge represent entry-points for social intervention, which reduce solid fuel use indirectly over long periods of time. A switch to cleaner fuels represents an entry-point for environmental intervention, which directly and immediately addresses hazardous household energy practices. An alternative would be promotion of cleaner-burning, more fuel-efficient stoves to reduce indoor air pollution, as demonstrated by a randomized controlled trial in Guatemala [96] and a systematic review of the effectiveness of different improved stove models in reducing household air pollution (Bruce et al., manuscript in preparation). As many projects and programs to improve access to modern household energy have either failed to reach the lowest-income group (e.g. Indonesian national LPG program [97]) or failed to bring about sustainable benefits (e.g. Indian National Improved Cookstove program [98]) we believe that technical interventions to reduce solid fuel use must be embedded in an integrated approach that considers and, to some extent, addresses socio-economic determinants if they are to benefit those who need them most. This conclusion accords with the findings of a recent systematic review of enablers and barriers to uptake of improved stoves [61].

Importantly, Figure 7 exposes neglected areas of research and can thus be useful in setting the research agenda. For example, it is noteworthy how few of the proximal risk factors are actually measured in DHS/WHS and that, based on routinely available data, no analysis of the multiple determinants of ALRI morbidity is feasible. Apart from further research along the lines reported here using additional datasets, efficacy and effectiveness trials of specific technical interventions, and natural experiments relating to policy changes, could illuminate the interrelationship of socio-economic conditions, solid fuel use and ALRI. The design of research could also benefit, e.g. by ensuring that omitted variables (residual confounding) are kept to a minimum, and, where possible, that datasets refer to a section of the diagram that can be disaggregated using conditional independence.

## Conclusions

We have described the use of causal diagrams as a means of organizing the current state of knowledge about a specific area of research, and as a framework for integrating statistical analyses across a whole system. The structure and content of the causal diagram is originally conceptualized using information from previous studies, and supplemented with assumptions and theories about how individual components of the system combine. This conceptualization of reality is then tested against observed data, using multiple datasets and data sources. Importantly, this method does not aim to decipher one-to-one relationships whilst holding all other variables constant; it is designed to understand whether and how components within the system work together and how these jointly promote or prevent disease.

We believe that Diagram-based Analysis of Causal Systems is well-suited to dealing with large and complicated systems, as long as they do not have feedback loops. While the combination of *a priori* hypotheses and their testing against observed data is common in many other scientific disciplines, such as environmental science, genetics and astronomy, it is still rare in epidemiology and the health sciences. We hope that this paper, together with recent commentaries calling for a shift in methodological focus in epidemiology [2,99,100], will help to raise awareness of the potential for systems approaches in epidemiology to account for the complexity of disease causation in populations.

We have applied our approach to explore the determinants of childhood ALRI in sub-Saharan Africa, an area of research that is characterized by limited data availability and poor data quality. These challenges are representative of many other neglected health problems in the developing world. The unique combination of rigor (in terms of comprehensive causal thinking) and flexibility (in terms of accommodating multiple sources of evidence) thus make Diagram-based Analysis of Causal Systems significant for public health research far beyond the research area considered here. The strength of this paper lies in the development of a protocol for developing and testing causal diagrams, and we invite those concerned with multifactorial health problems in the developing or developed world to test and improve our approach.

## Abbreviations

AIC: Akaike information criterion; ALRI: Acute lower respiratory infections; DHS: Demographic and Health Surveys; WHS: World Health Survey.

## Competing interests

The authors declare that they have no competing interests.

## Authors’ contributions

MJ’s expertise with causal diagrams was the starting point for the approach, which gradually evolved as part of ER’s PhD. ER developed and tested the approach with regular methodological and conceptual input from MJ, NB and DB. ER and MJ jointly drafted the manuscript, which was reviewed and critically revised by NB and DB. All authors read and approved the final manuscript.
